# Maximum Entropy Methods for Loss Data Analysis: Aggregation and Disaggregation Problems

**DOI:** 10.3390/e21080762

**Published:** 2019-08-06

**Authors:** Erika Gomes-Gonçalves, Henryk Gzyl, Silvia Mayoral

**Affiliations:** 1 Independent Consultant, 28014 Madrid, Spain; 2 Centro de Finanzas, IESA, Caracas 1010, Venezuela; 3 Department of Business Administration, Universidad Carlos III de Madrid, 28903 Getafe-Madrid, Spain

**Keywords:** loss data analysis, loss data aggregation, loss data disaggregation, operational risk, credit risk, sample dependence of loss distributions, sample dependence of risk premia, maximum entropy methods

## Abstract

The analysis of loss data is of utmost interest in many branches of the financial and insurance industries, in structural engineering and in operation research, among others. In the financial industry, the determination of the distribution of losses is the first step to take to compute regulatory risk capitals; in insurance we need the distribution of losses to determine the risk premia. In reliability analysis one needs to determine the distribution of accumulated damage or the first time of occurrence of a composite event, and so on. Not only that, but in some cases we have data on the aggregate risk, but we happen to be interested in determining the statistical nature of the different types of events that contribute to the aggregate loss. Even though in many of these branches of activity one may have good theoretical descriptions of the underlying processes, the nature of the problems is such that we must resort to numerical methods to actually compute the loss distributions. Besides being able to determine numerically the distribution of losses, we also need to assess the dependence of the distribution of losses and that of the quantities computed with it, on the empirical data. It is the purpose of this note to illustrate the how the maximum entropy method and its extensions can be used to deal with the various issues that come up in the computation of the distribution of losses. These methods prove to be robust and allow for extensions to the case when the data has measurement errors and/or is given up to an interval.

## 1. Introduction and Preliminaries

The problem of determining the distribution of aggregate losses begins by developing a model for the random variable in whose distribution we are interested. To state the basic problem, we suppose that all random variables involved are defined on a common probability space (Ω,F,P). A typical aggregate loss is modeled by a random variable like

(1) $$S=\sum_{h=1}^{H}S_{h}\;\;\;\mathrm{where}\;\;\;S_{h}=\sum_{n=1}^{N_{h}}X_{h,n}.$$

where H≥1 is some positive integer describing the total number of compound losses to be aggregated. For each loss type h, we shall suppose that $N_{h}$ is an integer-valued random variable modeling the frequency of losses in a given (and fixed) time lapse, and that for each h, the random variable $X_{h,k}$ is a positive, continuous random variable, modeling the occurrence of the individual *n*-th loss of that type. We shall follow the standard modeling assumption, and suppose that the individual losses are independent among themselves and independent of the corresponding $N_{h}.$

The standard “constructive direct” problem can be stated to consist of determining the probability density $f_{S}$ of the total loss *S* when we know the statistical nature of the $\{N_{h};H=1,\ldots ,H\},$ and that of the densities $f_{X_{h,n}}$ of the $\left\{X_{h,n}:h=1,\ldots ,H,;n\geq 1\right\}$. This is a “classical” problem that has been studied from many angles already reported in many treatises. When all that we have is empirical data about aggregate losses, and no models at hand, the direct approach may not be applied. In this case, we must recur to numerical procedures to determine the density of a continuous random variable *S* from an empirical sample $\{s_{1},\ldots ,s_{M}\}.$ The sample could be thought have been obtained by collecting data over a very large number of time lapses or to have been generated by simulating samples from the distributions that make up the building blocks of the model.

And this point of view connects us to a reciprocal related “inverse” problem. Suppose that we have a sample $\{s_{1},\ldots ,s_{M}\}.$ Besides recovering the density $f_{S},$ we want to recover the statistical information about the building blocks entering (1). That is, we want to know the statistical nature of the $N_{h},$ and as much as possible about the statistical nature of the $X_{h,n}.$

Since the probability density of a positive random variable can be recovered from its Laplace transform, here we examine how maximum entropy methods invert numerically the Laplace transform. What makes the use of the maximum entropy-based method important is that in most cases of practical interest, the Laplace transform must be estimated numerically. it is important that the inversion method is stable with respect to changes in the available data set and in the presence of errors in the data. This makes the maximum entropy methodology doubly interesting: it is robust and allows for extensions to incorporate errors in the data. We shall examine all these issues below.

Recall that the Laplace transform of (the density of) a continuous random variable *S* is defined by

(2) $$\mu \left(\alpha \right)=\int_{0}^{\infty }e^{-\alpha s}f_{S}\left(s\right)ds\;\;\;\mathrm{for}\;\;\;\alpha >0.$$

As mentioned above, when this quantity can be obtained from a model of S, and when it can be inverted, analytically or numerically, we are done. However, not only is it not always possible as in our examples, but in actual practice, what we may have is just empirical data. Thus, we must determine the Laplace transform from it, and then invert the estimated Laplace transform.

To bring in our proposed maxentropic methodology, it is convenient to perform the change of variables $y=e^{-s}$ and change problem (2) into a problem on [0,1], consisting of determining a density *g* from its fractional moments, i.e., to solve

(3) $$\int_{0}^{1}y^{\alpha }g\left(y\right)dy=\mu \left(\alpha \right)$$

where $f_{S}\left(s\right)=e^{-s}g\left(e^{-s}\right).$ It is at this point that the method of maximum entropy comes in.

We have already introduced enough notation to explain what comes next. The first issue to take care of is how to go from aggregate data such as (1) to estimate the right-hand side of (3). This is done in Section 2, Section 3 and Section 4 we shall rapidly review the standard maximum entropy (SME) method. The SME is the stepping stone for the extension to the case in which there are measurement errors (SMEE) and to the method of maximum entropy in the mean (MEM). These extensions are briefly described in the Appendix A.

In Section 5 we shall then examine how to relate the variability of the solution to (2) to the variability in the data, which comes through in the dependence of the μ(α) on the sample data, and in Section 6 we shall explain how the sample dependence of the reconstructed densities affects expected values computed with it. This is a rather important issue, because many quantities of interest depend heavily of the tail behavior of the density and cannot be estimated with a small amount of data in the tail of the distribution, if it exists at all. In the last Appendix A we add some further mathematical details about this issue.

After that, we shall explain why only a few moments are enough to solve (3) for g(y). This fact shows the power of the maxentropic procedure compared to the actual analytical inversion of (2) which requires the knowledge of the behavior of μ(α) in the complex α-plane.

In Section 8 we present some numerical examples. We begin by finding the density of a Poisson compound variable in which the individual losses are log-normal. As there is no closed analytical expression for the Laplace transform of such variables, the example stresses the need for the purely numerical approach. Then we tackle the problem of decompounding at the first level of aggregation.

After that, to illustrate the problem of disaggregation and decompounding, we consider data coming from two compound random variables. The same data will be used to illustrate the sample dependence of the density reconstruction and that of the sample dependence of risk measures (VaR and TVaR). Then, we shall illustrate the sample variability of some risk premia that depend strongly on the tail of the distribution.

## 2. The Aggregation Problem: From Data to Fractional Moments

In the process of data aggregation problems, there are several layers of aggregation, as implied in (1). In the first one, we have to form the compounded sums. The passage from the first layer of aggregation to the second involves computing the distribution of a sum of compound random variables. When there is statistical dependence between the variables, we must devise methods to take this into account. For details see [1,2].

Let us now explain how to go from the loss data to the fractional moment problem via the Laplace transform. It is well known—see [3] for examples—that the distribution function of a positive random variable *S* can be recovered from its Laplace transform, defined by

(4) $$\psi \left(\alpha \right)=E\left[e^{-\alpha S}\right]$$

Notice that the full distribution of *S* includes a point mass at S=0 which corresponds to the possibility of with no losses, which occurs when N=0. Therefore, to relate ψ(α) to the density of the continuous part of S, we must condition the mass at {S=0}. Notice that

$$E\left[e^{-\alpha S}\right]=P\left(S=0\right)+\left(1-P\left(S=0\right)\right)\int_{0}^{\infty }e^{-\alpha x}f_{S}\left(x\right)dx$$

and then

(5) $$\mu \left(\alpha \right)=\frac{\psi (\alpha )-P(S=0)}{1-P(S=0)}=\int_{0}^{\infty }e^{-\alpha x}f_{S}\left(x\right)dx.$$

where $f_{S}\left(x\right)=dP\left(S\leq x|S>0\right)/dx.$

We mentioned in the introduction that if we have a statistical model for the blocks of S, we can compute ψ(α) and from it μ(α), which is the starting point to compute $f_{S}.$ However, if we cannot compute ψ(α) analytically, which happens when, for example, the individual losses are log-normal, or when we only have observational data, we are forced to estimate μ(α) from a sample.

Therefore, if we record the losses for a certain number *N* of periods, in *M* of which we have a sequence like $\{n;x_{1},\ldots ,x_{n}\},$ where *n* is the number of risk events during the observation period, and the $x_{j},\;j=1,\ldots ,n$ are the losses occurring at each event, the total loss during the period is $\sum_{j=1}^{n}x_{j}.$ In the remaining N−M we observe no losses. Invoking the law of large numbers, we verify that an unbiased estimator of ψ(α) is

(6) $$\hat{\psi }\left(\alpha \right)=\frac{1}{N}\sum_{k=1}^{N}e^{-\alpha s_{k}}$$

Since the estimator of P(S=0) is (N−M)/N and it is clear that in this case, after some simple arithmetic, instead of (5), an estimator of μ(α) is given by:

(7) $$\hat{\mu }\left(\alpha \right)=\frac{1}{M}\sum_{k=1}^{M}e^{-\alpha s_{k}}.$$

These estimated moments will become the fractional moments that the SME method needs as inputs for the determination of the densities of losses. We also emphasize that it is through these estimated moments that the sample variability of the reconstructed densities comes in.

## 3. The Decompounding and Disaggregation Problems

We have already mentioned that the problem of decompounding is reciprocal to the problem of compounding. In the latter, we have to find the distribution function of a compound random variable. In the former, we are given aggregate data, and we are interested in the distribution of the individual severities. The reason for that is that the distribution of individual severities might be related to the cause of the losses, and its knowledge might be useful for control or preventive management. When the model of individual severities is known beforehand, the determination of individual severities provides a consistency check.

In the first aggregation level, the total severity is a compound variable, and the Laplace transform technique yields a direct connection between the Laplace transform of all the blocks of the model. The relationship is given by

(8) $$\psi \left(\alpha \right)=E\left[e^{\alpha S}\right]=G_{N}\left(E_{X}\left[\phi \left(\alpha \right)\right]\right)$$

where $G_{N}\left(z\right)=\sum_{n=0}^{\infty }P\left(N=n\right)z^{n}$ is the generating function of the frequency of losses and $\phi \left(\alpha \right)=\int_{0}^{\infty }\exp\left(-\alpha x\right)f_{X}\left(x\right)dx$ is the Laplace transform of the density of individual losses. Thus, once we know the law of N, we can solve ϕ(α) from (8) and then apply the SME method to obtain $f_{X}.$

Nice as this might be, it is only partially useful. The reason is that we might not have statistical information about the frequency of losses. A similar problem appears when the total loss has several levels of aggregation. Fortunately, in most cases the model is such that determining the statistical nature of the underlying frequency of events reduces to a problem of disentangling a collection of overlapping linear regressions, a problem which can be solved by a variety of methods. It is here that the material in the next section is essential.

### 3.1. The Panjer (a,b,0) Class

There is a particular class of frequency models for which determining the distribution of losses can be carried out systematically. Not only that, but also knowing that the frequencies belong to this class makes the disentangling of the frequencies a routine matter.

Please note that the probabilities p(n)=P(N=n) of the binomial, geometric, negative binomial, and Poisson distributions satisfy the recursion relation p(n)/p(n−1)=a+b/n. See [4] for details.

There k,ℓ,r,n∈N, and β∈(0,∞).

If we set $r\left(k\right)=p_{k}/p_{k-1},$ we can write the recursion in Table 1 as kr(k)=ak+b, which can be thought of as linear regressions. If we empirically estimate r(k) by $\hat{r}\left(k\right)$ and plot $\left(k,\hat{r}\left(k\right)\right)$ we can use the plot to obtain the coefficients (a,b) and then the parameters of the distributions as specified in Table 1. Not only that, but also if we have a collection of risks such that their collective frequency can be considered to be a mixture of the frequencies of the collection, we can use the representation provided by the Panjer recursions plus a clustering or classification technique to determine the underlying frequency models. This will be the starting point of the decompounding procedure implemented in the section of numerical results. This procedure is detailed in [1] and later in [2]; we direct the reader there for complete details and many references to the subject.

## 4. The Maxentropic Solution of the Fractional Moment Problem

Above, we explained how to transform the Laplace inversion problem into a fractional moment problem consisting of finding a probability density g(y) on [0,1] satisfying the integral constraints:

(9) $$\int_{0}^{1}y^{\alpha_{k}}g\left(y\right)dy=\mu \left(\alpha_{k}\right)\;\mathrm{for}\;k=0,1,\ldots ,K.$$

where the $\mu (\alpha_{k})$ are estimated as in (7). At this point two questions come up: first, do the fractional moments determine uniquely the distribution? And then a practical question: how to actually solve (9).

The answer to the first is resolved in the positive in [5]: the fractional moments determine uniquely a density g. A method to solve the (9) problem seems to have been originally proposed in [6] or [7], and its mathematical subtleties are addressed in [8]. This method can be extended in several directions. Here we shall consider two possible extensions. One of them, the standard maximum entropy with errors in the data (SMEE), is particularly useful when the sample dependence is strong and can be regarded as additive noise. The other method, that of MEM, is an important alternative maxentropic method, particularly useful when there are convex constraints imposed on it.

In (9), we set $\alpha_{0}=0$ and $\mu \left(\alpha_{0}\right)=\mu_{0}=1$ to take care of the natural normalization requirement on g(y). The intuition behind the maxentropic methods is rather simple: The class of probability densities satisfying (9) is convex. One can pick up a point in that class one by maximizing (or minimizing) a concave (convex) functional (an “entropy”) that achieves a maximum (minimum) in that class. That optimal point is the “maxentropic” solution to the problem. It actually takes a standard computation to see that when the problem has a solution, it is of the type

(10) $$g\left(y\right)=\exp\left(-\sum_{k=0}^{K}\lambda_{k}^{*}y^{\alpha_{k}}\right)$$

which depends on the αs through the λs. It is usually customary to write $e^{-\lambda_{0}^{*}}=Z{\left(\lambda^{*}\right)}^{-1},$ where $\lambda^{*}=\left(\lambda_{1}^{*},\ldots ,\lambda_{K}^{*}\right)$ is a *K*–dimensional vector. Clearly, the generic form of the normalization factor is given by

(11) $$Z\left(\lambda \right)=\int_{0}^{1}e^{-\sum_{k=1}^{K}\lambda_{k}y^{\alpha_{k}}}dy.$$

With this notation the generic form of the solution looks like

(12) $$g^{*}\left(y\right)=\frac{1}{Z(\lambda^{*})}e^{-\sum_{k=1}^{K}\lambda_{k}^{*}y^{\alpha_{k}}}=e^{-\sum_{k=0}^{K}\lambda_{k}^{*}y^{\alpha_{k}}}.$$

Recall that once $g^{*}\left(y\right)$ is at hand, the density on the half line is given by $f_{S}^{*}\left(s\right)=e^{-s}g^{*}\left(e^{-s}\right).$ How to determine $\lambda^{*}$, and further details about the SME procedure are presented in the Appendix A.

We close this section stating a result that plays a role in the forthcoming sections. It takes a standard computation to verify that

(13) $$S\left(f^{*}\right)=\ln Z\left(\lambda^{*}\right)+\langle \lambda^{*},\mu \rangle .$$

## 5. Variability of the Probability Densities

The results presented next are a variation on the theme of sample dependence in the generalized moment problem studied in [9] and applied in [10]. The necessary results are stated without proof and we direct the reader to [9] for details and proofs.

Denote by $\mu_{M}\left(\alpha \right)$ the moments computed as in (7) and $\mu_{e}\left(\alpha \right)$ the exact moments. Denote as well $\lambda_{M}^{*}$ and $\lambda_{e}^{*}$ the corresponding minimizers of (A5) and by $f_{M}\left(x\right)$ and $f_{e}\left(x\right)$ the maxentropic densities.

**Lemma** **1.** *With the notations just introduced, suppose that the risks observed during M consecutive years are independent of each other.*

Then $\mu_{M}\left(\alpha \right)\to \mu_{e}\left(\alpha \right)$ and $\lambda_{M}^{*}\to \lambda_{e}^{*},$ and therefore $f_{M}\left(x\right)\to f_{e}\left(x\right)\;\;\mathrm{when}\;\;M\to \infty .$

To relate the sample variability of $f_{M}$ to the sample variability of the μ(α), we start with $\lambda_{M}^{*}=\phi^{-1}\left(\mu \right),$ and apply the chain rule, to obtain that up to terms of o(δμ)

(14) $$\delta \lambda_{M}^{*}=D\delta \mu$$

where D is the inverse matrix of the Jacobian of $\phi \left(\lambda \right)=-\nabla_{\lambda }\ln Z\left(\lambda \right)$ evaluated at $\lambda_{M}^{*}.$ Recall that the maxentropic solution to the inverse problem is

$$f_{M}\left(y\right)=\frac{1}{Z(\lambda_{M}^{*})}e^{-\sum_{k=1}^{K}\lambda_{k}^{*}y^{\alpha_{k}}}$$

in which reference to the sample size *M* is made explicit. Consider now:

**Lemma** **2.** *With the notations introduced above, up to terms that are o(δμ), using the computation in (14)*

(15) $$f_{M}\left(x\right)-f_{e}\left(x\right)=\sum_{i,j=1}^{K}\left(\mu \left(\alpha_{i}\right)-e^{-x\alpha_{i}}\right)f_{e}\left(x\right)D_{i,j}\delta \mu_{j}.$$

*Here K is the number of moments used to determine the density and $\delta \mu_{j}=\mu_{M}\left(\alpha_{j}\right)-\mu_{e}\left(\alpha_{j}\right).$*

This result relates the deviation of $f_{M}$ from its exact value $f_{e}$ up to first order in the δμ. Notice if we integrate both sides of (15) with respect to *x* we get 0 on both sides. In addition, if we multiply $f_{M}\left(x\right)$ and $f_{e}\left(x\right)$ by $e^{-x\alpha_{k}},$ we obtain $\delta \mu_{k}.$

Using this, Proposition A1 in the Appendix A, and Lemma 1, the following is clear.

**Proposition** **1.** *With the notations introduced above we have*

(16) $$∥f_{M}-f_{e}{∥}_{1}\leq {\left(4K(f_{e},f_{M})\right)}^{1/2}\to 0\;\;as\;\;M\to \infty .$$

We direct the reader to Appendix 10.3 where K(f,g) is defined and its properties stated. We can also combine Lemma 1, (A9) and (1) to relate the deviations $∥f_{M}-f_{e}{∥}_{1}$ to the deviations of $\mu_{M}$ from $\mu_{e}$ as described in the following result.

**Proposition** **2.** *With the notations introduced above we have*

(17) $$∥f_{M}-f_{e}{∥}_{1}\leq {\left(2\langle \delta \mu ,D\delta \mu \rangle \right)}^{1/2}\to 0\;\;as\;\;M\to \infty .$$

*Here δμ and D are as above.*

The proof drops out from the fact that up to terms of order $o({\left(\delta \mu \right)}^{2}),$$K\left(f_{e},f_{M}\right)=\langle \delta \mu ,D\delta \mu \rangle .$

## 6. Sample Variability of Expected Values

Before describing the sample variability of expected values, we address an obvious question. Notice that the determination of any risk premium involves the computation of an expected value with respect to a density of losses. For a review of premium calculation procedures see [11,12]. It is here where the results described in Section 5 come in. An obvious issue comes to mind. If we need to compute E[h(S)] for some positive, measurable h(x) and all we have is sample data $\{s_{1},\ldots ,s_{N}\},$ why not just compute $\sum_{i\geq 1}h\left(s_{i}\right)/N$? And if we are interested in the sample variability, we might as well just look at the variability of

(18) $${\hat{h}}_{N}=\frac{1}{N}\sum_{i=1}^{N}h\left(S_{i}\right)$$

whose statistical properties are standard procedure, i.e., if we consider the sample values $h(S_{i})$ as a collection of i.i.d. random variables, their confidence intervals, the oscillations about the mean, and the rate of convergence to the mean can be treated using standard statistical tools. However, if we happen to be interested in the computations of quantities such as

(19) $$\pi \left(S\right)=E\left[\min\left(K,{\left(S-d\right)}^{+}\right)\right]=\int_{d}^{K+d}\left(s-d\right)f_{N}\left(s\right)ds+K\int_{K+d}^{\infty }f_{N}\left(s\right)ds.$$

in which the value *d* may be of the order of the 90% quantile of *S* and *K* may be larger, the dependence on the sample becomes harder to handle because we would have to deal with values at the tail of the distribution. Such problems do not appear if we compute expected values with respect to a density whose dependence on the sample can be made explicit.

As with the case of Lemma 1, an appeal to the law of large number yields the simple observation.

(20) $$|{\hat{h}}_{N}-E_{N}\left[h\left(S\right)\right]|\to 0,\;\;\;\mathrm{as}\;\;\;N\to \infty .$$

The sample variability of expected values, and in particular, for quantities such as (19), follows from the following generic result which is a direct consequence of Lemma 1.

**Lemma** **3.** *Let us denote Let h:[0,∞)→R be such that $E_{e}\left[|h\left(S\right)|\right]<\infty .$ Then*

$$\int h\left(S\right)f_{N}\left(x\right)dx=\int h\left(S\right)f_{e}\left(x\right)dx+\int h\left(x\right)\left(\sum_{i,j}\mu \left(\alpha_{i}\right)-e^{-x\alpha_{i}}D_{i,j}\delta \mu_{j}\right)f_{e}\left(x\right)dx,$$

*where the randomness is carried by the second term on the right-hand side. Clearly,*

$$|\int h\left(S\right)f_{N}\left(x\right)dx-\int h\left(S\right)f_{e}\left(x\right)dx|\leq \int |h\left(x\right)|\sum_{i,j}\left|\mu \left(\alpha_{i}\right)-e^{-x\alpha_{i}}D_{i,j}\delta \mu_{j}\right|f_{e}\left(x\right)dx.$$

The relevance of this Lemma is that it relates the deviations of the estimated densities from the true density to the deviations of the empirical moments from the true moments. Similar considerations apply to the computation of risk measures lake VaR and TVaR, which depend of the tail of the distribution. A relationship between them is established in [13]. In Section 8.6 and Section 8.7 we present some numerical examples of sample dependence of risk measures and premium prices.

## 7. Number of Moments Necessary to Determine the Density

Here we address a problem of practical interest: How many values of the Laplace transform parameter α are necessary to determine $f_{S}.$ This is interesting in itself because the theoretical inversion of a Laplace transform involves an integration in the complex α plane, which in principle requires the knowledge of an extension of μ as an analytic function and the location of its singularities in the complex plane. There are inversion methods based on the knowledge of μ(α) on the positive half of the real line (see [3] for an introduction to this subject).

Here, we only remark that the maximum entropy method needs a few points to work (for us 8 values were enough). The explanation of why this seems to be so goes as follows. Let us denote by $f_{M}^{*}$ the maxentropic density estimated from *M* moments. It is easy to see from (A8) and (13) that $K\left(f_{M}^{*},f_{M+1}^{*}\right)=S\left(f_{M}^{*}\right)-S\left(F_{M+1}^{*}\right)>0.$ In addition, from the inequality stated in Proposition A1 we have

$$\frac{1}{4}{∥f_{M}^{*}-f_{M+1}^{*}∥}_{1}^{2}\leq \left(S\left(f_{M}^{*}\right)-S\left(F_{M+1}^{*}\right)\right).$$

Thus, if a density with the given sequence of moments exits, and has finite entropy, the sequence $S(f_{M}^{*})$ is bounded below and the previous inequality assets that the densities converge (to the true density). In addition, usually, for 4 moments the sequence of entropies tends to stabilize. See [14] for more on this.

## 8. Numerical Examples

Below we present some numerical examples of the different problems that can be solved with maximum entropy-based methodology. We begin by considering a first level of aggregation and disaggregation process that is the problem of determining the distribution of a compound random variable form numerical data, and then how to go from the numerical data to fit a model for the frequency of compounding and the distribution of the individual severities. Next, we do the same for the case in which we need to aggregate or disaggregate several compound variables. In these examples we do the density reconstruction using the SME and the SMEE methods. When the data is obtained from a sample, we expect the SMEE to outperform the SME.

In each case, we carry out the corresponding reconstruction error estimation. After examining these examples, we consider the issue of sample variability and its effect, not only in the density reconstruction process, but in the estimation of quantities of interest, like risk measures (VaR and TVaR) or risk premia, among others.

All the code, with the examples used in this article can be obtained from [15]. The results presented there may change due to convergence of the algorithm, choice of seeds, etc.

### 8.1. The Sample Generation Process

We consider first a total severity at the first level of aggregation consisting of compounding individual log-normal severities with a Poisson frequency. The parameters to use are ℓ=4 for the Poisson distribution and μ=6 and σ=0.5 for the log-normal distribution.

After that we shall consider a second level of aggregation, consisting of a sum $S=S_{1}+S_{2},$ of two compound variables, in which the first ($S_{1}$) is the one we just finished describing, and the second ($S_{2}$) is a Poisson (with ℓ=8,) compound sum of individual severities described by a Gamma with shape parameter a=350 and scale parameter b=3.

In these examples we consider losses of an order of magnitude of $10^{3},$ and to avoid numerical overflow or underflow, we scale down these values, and ,at the end, we scale back the reconstructed density to its original units. We do this for all the examples described here. Here, we work with three different sample sizes of 100, 500, and 2000.

The sample of size 500 will be used for validation purposes. We shall use the densities obtained from the samples of sizes 100 and 2000 to see how well they fit the data of the sample of size 500.

#### Error Measurement

To finish the preamble, we mention that to measure the discrepancy between the estimated density and the histogram, we used the Mean Absolute Error (MAE) and the Root Mean Squared Error (RMSE). These are computed as follows

$$MAE=\frac{1}{N}\sum_{j=1}^{N}\left|F_{S}^{*}\left(s_{j}\right)-F_{N}\left(s_{j}\right)\right|$$

$$RMSE=\sqrt{\frac{1}{N}\sum_{j=1}^{N}{\left(F_{S}^{*}\left(s_{j}\right)-F_{N}\left(s_{j}\right)\right)}^{2}}$$

A nice feature of these error measures is that they compare the estimated distribution function $F_{S}^{*}$ and the cumulative distribution function $F_{N}$ at the sample data only. See [16] for further details.

### 8.2. Density Reconstruction

As a first example, we consider the determination of the density of a loss at the first level of aggregation. We consider the Poisson compound sum of log-normal random variables described above. To stress the point once more, this density was chosen for two reasons. First its analytic form is known, therefore it serves as reference to compare the densities obtained by the maximum entropy method at the decompounding stage. Second, its Laplace transform cannot be computed analytically, and we must resort to simulated data.

**Figure 1:**
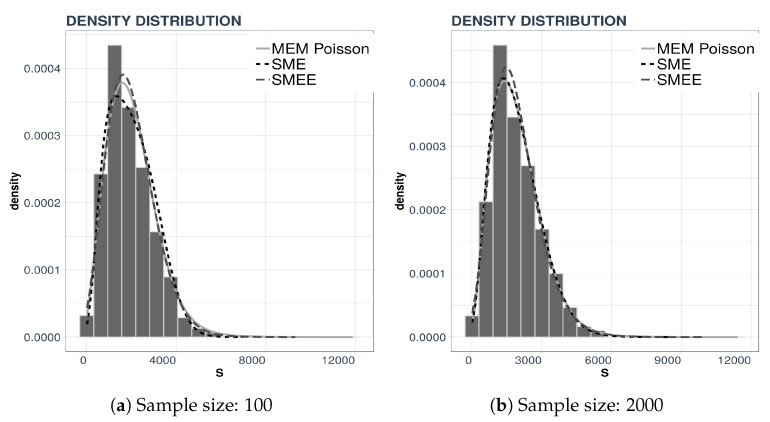
Density reconstruction using three entropy-based methods.

For the example we consider two data sets, consisting of either 100 or 2000 data points and perform a density reconstruction with the three maxentropic techniques: SME, SMEE, and MEM. The results are displayed in Figure 1 along with the corresponding data histograms.

In each panel we present three curves. In the left panel, Figure 1a we present the density reconstructed by the three maxentropic methods using 100 data points, whereas in the right panel, Figure 1b, we present the reconstruction from 2000 data points. Consider Table 2—there, one can see that the standard maxentropic reconstruction with errors in the data yields the best fit.

Two important quantities for risk management and regulatory capital determination are the VaR and the TVaR. These can be readily computed once the density (or cumulative distribution functions) are at hand. In Table 3 below, we list these quantities for the distribution obtained by the three methods.

#### A Validation Procedure

We carry out a validation procedure proposed in [17] under the name “Calibration Diagram”. The validation consists of a visual comparison of plots. In our case, the cumulative empirical distribution of a data set of 500 is the test distribution. We then compare it to the cumulative densities obtained from 100 or 2000 data points by plotting the difference $F_{S}^{*}\left(s_{j}\right)-F_{500}\left(s_{j}\right),$ where $F_{S}^{*}$ is the cumulative distribution function of *S* obtained by the maxentropic methodology from either 100 or 2000 data points. This test tells us how well the densities from large or small data sets predict or fit data sets of a different size.

**Figure 2:**
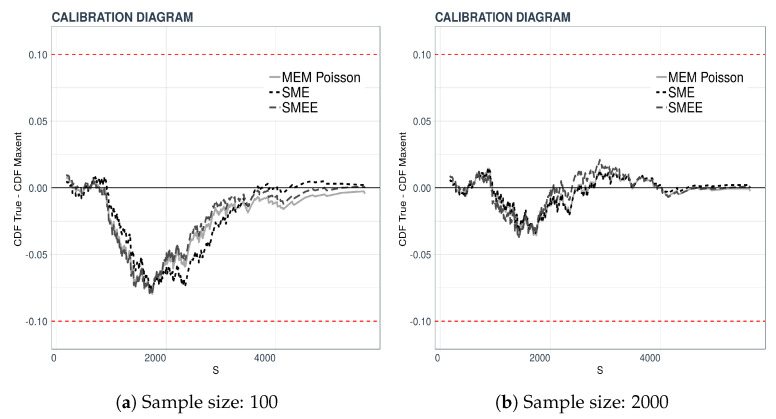
Results of the validation test.

The results of the validation procedure are displayed in Figure 2. In the left-hand panel, the maxentropic densities were obtained from the sample of size 100 and those on the right from the full sample of size 2000, and, as said, the empirical distribution is that of a sample of size 500. Clearly, the larger difference between the distributions occurs in the central region of the range of the variable.

### 8.3. Decompounding at the First Level of Aggregation

Here we shall consider an implementation of the theme described in Section 3. We already noted that the Laplace transform of a compound sum of positive random variables can be written as $\psi \left(\alpha \right)=G_{N}\left(\phi (\alpha )\right),$ where ϕ(α) is the Laplace transform of the individual severity. We know how to compute ψ(α) from a sample, and if we can solve the last equation for ϕ(α), we can then apply the maxentropic machinery to obtain the density of the individual severity.

We already saw in Section 3 that when the frequency of losses is in the Panjer (a,b,0) class, there is a systematic way to obtain the model for the loss frequency. This was carried out in [1,2], so let us not repeat it here. According to the Panjer plot, the distribution seems to be Poisson. Even though this can be double-tested compared its mean and its variance, we use the following indirect approach to find the correct value of ℓ.

**Figure 3:**
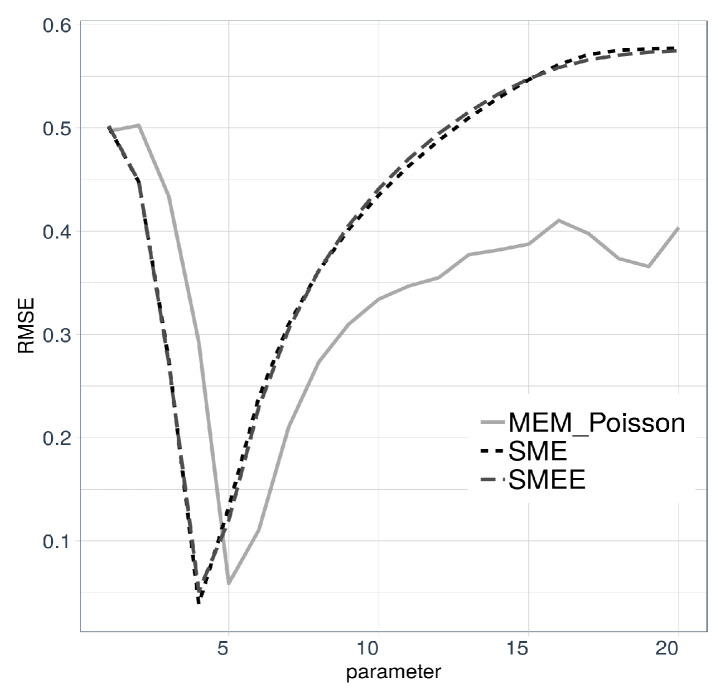
Values of RMSE for different values of parameter *ℓ*.

Therefore, let us suppose that the frequency model is Poisson, and solve for ϕ(α) in (8) to obtain ϕ(α)=1+ln(Ψ(α))/ℓ, where *ℓ* now stands for the unknown parameter of the Poisson distribution (which for the sake of comparison, we know to be λ=4). The quest for the best *ℓ* goes as follows. Fix *ℓ* and use maxentropic procedures to obtain $f_{X}^{*}.$ For each version of the maxentropic density, we compute the RMSE reconstruction error for each *ℓ* and eventually settle for the value of *ℓ* that yields a smaller reconstruction error. The results are displayed in Figure 3. For the SME and SMEE-based methods, the optimal Poisson parameter happened to be λ=4, whereas for the reconstruction using MEM, it happened to be λ=5.

**Figure 4:**
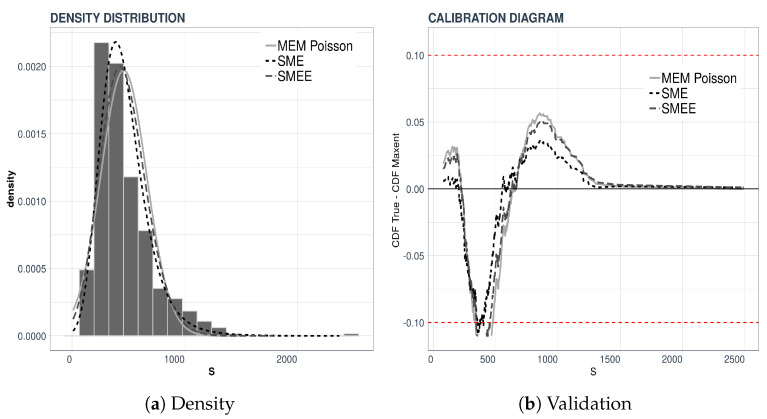
Individual severities using three entropy-based methods.

Once the value of *ℓ* is determined, we move on to the determination of the probability density of the individual severities. In Figure 4 we display the densities of the individual severities for the two sample sizes that we considered.

The left-hand panel of Figure 4a displays the reconstructions of the density of the individual severities when the Laplace transform of the total loss was computed from 2000 data points, whereas the right-hand panel, Figure 4b, displays the result of a validation test like the one explained above. This time we compared the densities obtained from 500 and 2000 data points.

Next we list the reconstruction errors in Table 4. As above, we use the MAE and RMSE as discrepancy error between the reconstructed densities, and the true density, which in our case happens to be known. This test cannot be performed in practice when all that is available to us is the aggregate data.

### 8.4. Decompounding and Disaggregating Risk Sources

When there are several sources of risk, to determine the possible distributions of individual losses becomes a truly hard problem. To begin with, the analysis requires an initial step consisting of disentangling the possible underlying frequencies of events. For this we must estimate how many sources of risk are present in the data and which are their corresponding frequency of events. After this comes the determination of the distributions of individual severities. It turns out the best we can do here is to recover an appropriate weighted mixture of the individual loss densities. This step is essentially the same as that developed above for the single risk source case. We direct the reader to [2] for further details and more elaborate examples.

There are several methodologies that can be used to determine the number of sources present in the data, such as AIC, AIC3, BIC, ICL-BIC, and Negentropy, all discussed in [2]. The essential assumption is that the frequencies of events are in the Panjer (a,b,0) class. This transforms the problem into a problem consisting of disentangling a collection of linear regressions.

**Figure 5:**
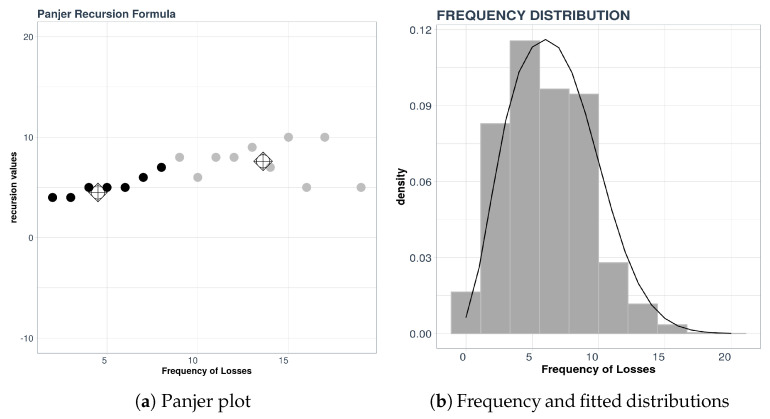
Frequency distribution and K-means result.

For the example considered here, consisting of the aggregate data described above, we will use the Panjer plot technique combined with a k-means clustering procedure. In Figure 5, we see the distribution of the frequencies (which is a mixture of distributions) and the result of applying the Panjer recursion formula and k-means.

In Figure 5a we see that there appears to be two groups of points with zero slope; one group is centered around the value 4 and the other group is centered around the value 8 on the vertical axis. These two values represent the parameters of the distribution, which is the Poisson distribution as indicated in Table 1 of Section 3.1.

To test the quality of the results we compared frequency data with the density of the mixture determined by our procedure. The result is displayed in Figure 5b. The analysis is completed using other measures of statistical error. For details about this, consult [2], for example.

Once we have the parameters of the mixture of frequencies, we apply the decompounding procedure. Regretfully, the nature of the problem is such that the best one can do is to obtain an “equivalent” individual loss density which is a mixture of the true densities, or in other words, the random variable modeling the aggregate loss is a mixture of the random variables modeling the aggregate losses. The formal result in the case at hand is:

$$f_{\hat{X}}=\frac{E[N_{1}]}{E\left[N_{1}\right]+E\left[N_{2}\right]}f_{X_{1}}+\frac{E[N_{2}]}{E\left[N_{1}\right]+E\left[N_{2}\right]}f_{X_{2}}$$

where $f_{X_{1}}$ and $f_{X_{2}}$ are the probability densities of the individual severities that are unknown. As this is usually the case, by the use of the maxentropic methodology we can decompound the aggregate losses as before to obtain the distribution of the individual severities that produces the observed aggregate loss.

### 8.5. Sample Size Dependence of the Reconstructed Densities

To examine the sample size dependence of the densities obtained by the maxentropic method, we consider sample sizes of 10, 20, 50, 200, 500, and 1000 of a total loss resulting from aggregating to compound losses that is $S=S_{1}+S_{2}$. We supposed as well that the two compound losses $S_{1}$ and $S_{2}$ are independent. In [18] we go through various elaborate schemes of coupling to analyze the aggregation issue.

**Figure 6:**
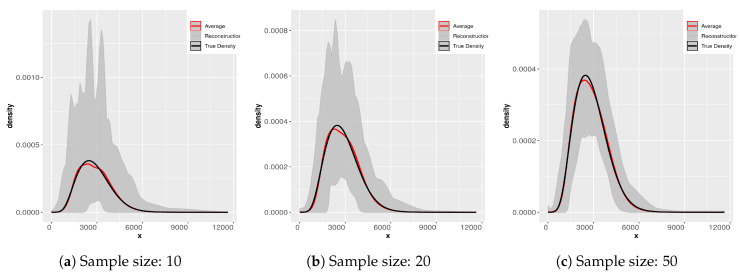
Densities from samples of sizes 10, 20, 50.

**Figure 7:**
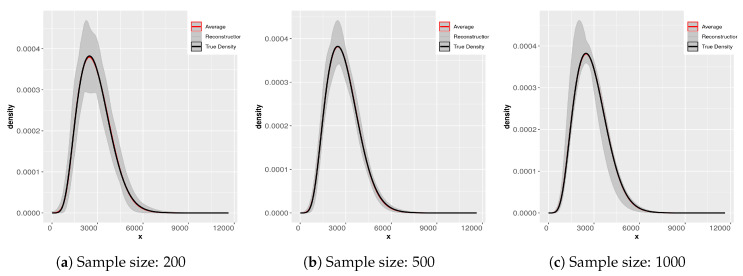
Densities from samples of sizes 200, 500, 1000.

In Figure 6 and Figure 7 we display the results of applying the SME methodology to 200 samples of each different sample size. Notice that the scale in the ordinate axis in all the plots is not the same. The subsamples were obtained from the original sample of size 2000. The gray-colored cloud is generated by simultaneously plotting all the densities for each sample size in a light gray line. The black line is that of the “true” density and the dashed line is the average of the 200 reconstructions. Notice as well that the average essentially coincides with the “true” density (reconstructed from the sample of size 2000). What changes from panel to panel is the variability of the reconstructions. Clearly for small sample sizes the variability is greater. We direct the reader to [9,10] for further analysis of this problem.

This exercise is useful for several reasons, when we do not have any information about the underlying process of the compound losses (i.e., about the statistical nature of the frequency and/or that of the individual severities), to try to fit a parametric distribution can be problematic. If the available sample is too small, we can have problems estimating the body and the tail of the distribution correctly, especially in the case of heavy tailed distributions. We display the result of the maxentropic procedure for different sample sizes to gain intuition about the quality of the results.

Since the maxentropic procedure yields a curve, we can examine how the reconstructed densities fit the body and the tail in the full range of values of the variable. It is also clear that the average of the reconstructions approximates the true density, so if you have enough data, resampling can help to improve the results of the methodology.

### 8.6. The Sample Size Variability of VaR and TVaR

**Figure 8:**
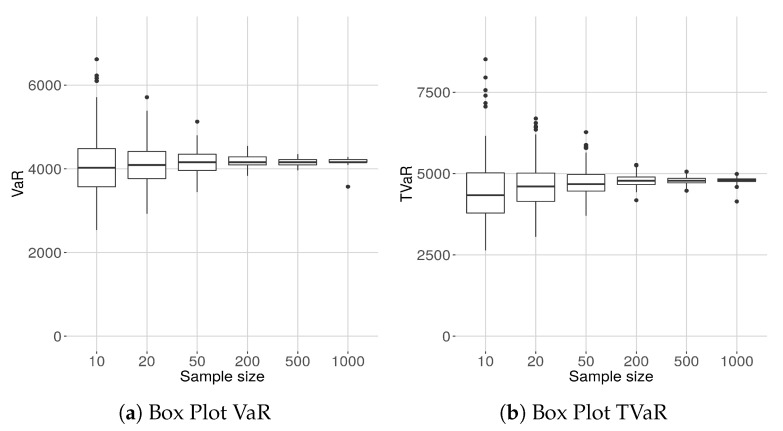
Sample size dependence VaR and TVaR at the 90% confidence level.

In Figure 8 we show the box plots of the estimated of VaR and TVaR for different sample sizes. The VaR and the TVaR are estimated from the densities plotted in Figure 7.

The results are intuitive, but worth keeping in mind, especially for risk-measurement purposes.

### 8.7. Sample Dependence of Risk Prices

In this section, we consider the computation of two risk prices that depend heavily on the knowledge of behavior of the density at the extreme tail of the distribution. Here we examine how the size of the sample data affects the computation of the premia. In each of the examples, we consider 200 subsamples of samples of sizes 10,20,50,200,500,1000. For each we compute each of the expected values and display the results as box plots.

Here $f_{N}^{*}\left(s\right)$ denotes the maxentropic density obtained from a sample of size N. We mention that similar results are obtained using SME or MEM.

#### 8.7.1. Stop-Loss with a Cap

We consider first the sample dependence of a risk a pricing procedure used in the insurance industry for the purpose of reinsurance policy pricing. For each of the 200 samples of size *N* we compute (19), that is

$$\pi \left(S\right)=E\left[\min\left(K,{\left(S-d\right)}^{+}\right)\right]=\int_{d}^{K+d}\left(s-d\right)f_{N}^{*}\left(s\right)ds+K\int_{K+d}^{\infty }f_{N}^{*}\left(s\right)ds.$$

using the maxentropic density using SME, and plot the results in a box plot diagram. For the computations, we used d=VaR(S) and K=TVaR(S) at the 90% level of confidence. Here $f_{N}^{*}\left(s\right)$ denotes the maxentropic density obtained from a sample of size N. We mention that similar results are obtained using SME or MEM.

#### 8.7.2. A *g*–Distorted Premium Price

For this example, we considered $g\left(x\right)=\left(1-e^{-u}\right)/\left(1-e^{-u}\right)$ as the distortion function. To obtain the risk price $\pi_{N}\left(S\right)$ we must apply (21), that is, we must compute

(21) $$\pi_{g}\left(S\right)=\int_{0}^{\infty }xg\left(F_{N}^{*}\left(x\right)\right)f_{N}^{*}\left(x\right)dx.$$

**Figure 9:**
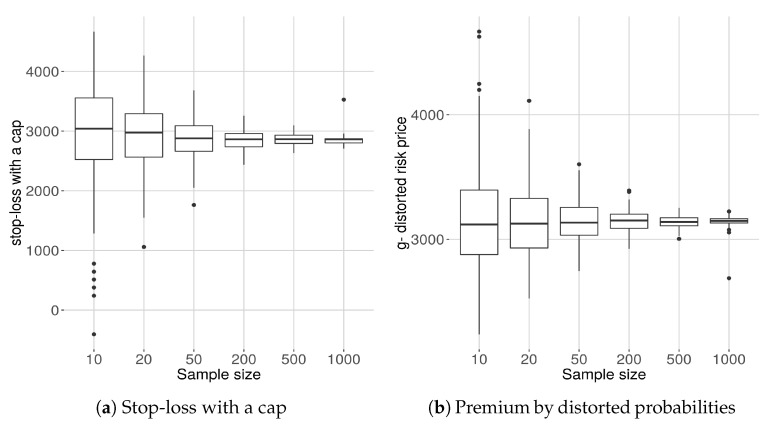
Box plots of premia for different sample sizes.

Of course, the computations were carried out numerically and as upper limit of integration we considered 1.5× (upper limit of sample). In Figure 9 we display the box plots of the premia computed using the maxentropic distribution with the Laplace transform computed from samples of different sizes. Even though the result agrees with our intuition, it is nevertheless important to have a quantitative idea of the range of variability of the possible values.

## 9. Concluding Remarks

Clearly, besides supposing that loses can be described as compound random variables, and that the individual losses are continuous, we have not made any other modeling assumptions. In other words, the methodology based on the maximum entropy method provides us with a truly model-free, non-parametric, and robust technique to obtain loss densities.

When the amount of data is moderately large, combining it with a resampling procedure, we can obtain quite trustworthy estimates of the loss densities, risk measures, and risk premia, or any other quantity expressed as expected value with respect the loss density. This last detail is especially important when the quantities if interest depend strongly on the tail of the density that is of values of the loss that may not be observed in the data.

As a matter of fact, as the last two examples show, even with reasonably large samples, there can be outliers present in the computation of risk measures and risk prices. This is an important detail for risk analysts to keep in mind.

## Figures and Tables

**Table 1:** Relationship between the parameters (a,b) and the discrete distributions.

Panjer Recursion $p_{n}/p_{n-1}=a+b/n;\;n\geq 1$				
Name	Distribution	*a*	*b*	*p*_0_
Poisson	$p_{n}=\frac{e^{-\ell }\ell^{n}}{n!}$	0	*ℓ*	$e^{-\ell }$
Binomial	$p_{n}=$ $\left(\begin{array}{c} m\\ n \end{array}\right)$ $q^{n}{\left(1-q\right)}^{m-n}$	$-\frac{p}{1-p}$	$\left(m+1\right)\frac{p}{1-p}$	${\left(1-p\right)}^{n}$
Neg. Binomial	$p_{n}=$ $\left(\begin{array}{c} n+r-1\\ n \end{array}\right)$ ${\left(\frac{1}{1+\beta }\right)}^{r}{\left(\frac{\beta }{1+\beta }\right)}^{n}$	$\frac{\beta }{1+\beta }$	$\left(r-1\right)\frac{\beta }{1+\beta }$	${\left(1+\beta \right)}^{-r}$
Geometric	$p_{n}=\left(\frac{1}{1+\beta }\right){\left(\frac{\beta }{1+\beta }\right)}^{n}$	$\frac{\beta }{1+\beta }$	0	${\left(1+\beta \right)}^{-r}$

**Table 2:** Errors in the different approaches. Case: ℓ=4, μ=6, σ=0.5.

Sample Size	Approach	MAE	RMSE
2000	SME	0.01194	0.01484
	SMEE	0.01315	0.01648
	MEM	0.01295	0.0155
100	SME	0.03619	0.0444
	SMEE	0.03530	0.04336
	MEM	0.03665	0.04413

**Table 3:** VaR and TVaR of the compound losses at the 95% confidence level.

Sample Size	Approach	VaR	TVaR
2000	SME	3643	4226
	SMEE	3710	4425
	MEM	3542	4189
100	SME	3701	4114
	SMEE	3808	4445
	MEM	3709	4416
2000	Sample	3762	4407

**Table 4:** Errors of the different approaches.

Sample Size	Approach	MAE	RMSE
2000	SME	0.04558	0.05729
	SMEE	0.05536	0.06805
	MEM	0.06395	0.0782

## Appendix A

In this section, we provide some extra theoretical details about the maximum entropy method, about the sample dependence, and about the necessary number of moments.

### Appendix A.1. Comments on the SMEE Method

Here we shall consider the following extension of problem (9). Notice that as the moments there are estimated from a sample, and we have two possible sources of error. On the one hand, there is the problem of the passage of the integral to a sum, and on the other the problem arises because the moments are computed from a sample. Thus, we must consider an inverse problem like

$$\int_{0}^{1}y^{\alpha_{k}}g\left(y\right)dy+\epsilon_{k}=\mu_{k},\;\;\;k=1,\ldots ,K.$$

The $\epsilon_{k}$ represents “measurement” errors. Observe that as the right-hand side is obtained as an average, it makes sense to think about the $\epsilon_{k}$ as the result of an average with respect to an unknown probability to be determined as well. To make things notationally simple, we shall suppose that $\epsilon_{k}\in \left[-d,d\right]$ where *d* might denote a confidence interval. In this case, we have $\epsilon_{k}=-dp_{k}+d\left(1-p_{k}\right)$ with respect to unknown $p_{k},k=1,\ldots ,K.$ Our problem now becomes: determine a probability on $\left[0,1\right]\times {\left[-d,d\right]}^{K},$ such that

(A1) $$\int_{0}^{1}y^{\alpha_{k}}g\left(y\right)dy-dp_{k}+d\left(1-p_{k}\right)=\mu_{k},\;\;k=1,\ldots ,K.$$

The variational method proposed by Jaynes goes as follows: define the entropy function by

$$S\left(g,p\right)=-\int_{0}^{1}g\left(y\right)dy-\sum_{k=1}^{K}p_{k}\ln p_{k}$$

which is a strictly concave function, and solve the problem: find a density $g^{*}\left(y\right)$ and numbers $0<p_{k}^{*}<1,k=1,\ldots ,K$ such that

(A2) $$\left(g^{*},p^{*}\right)=argsup\left\{S\left(g,p\right)\;:\;\mathrm{Subject}\mathrm{to}\;\left(A1\right)\right\}.$$

The solution to this problem is given by

(A3) $$\begin{array}{c} g^{*}\left(y\right)=\frac{1}{Z(\lambda^{*})}e^{-\sum_{k=1}^{K}\lambda_{k}^{*}y^{\alpha_{k}}}\\ \epsilon_{k}=\frac{-de^{d\lambda_{k}^{*}}+de^{-d\lambda_{k}^{*}}}{e^{d\lambda_{k}^{*}}+e^{-d\lambda_{k}^{*}}} \end{array}$$

The normalization factor Z(λ) is given by

(A4) $$Z\left(\lambda \right)=\int_{0}^{1}e^{-\sum_{k=1}^{K}\lambda_{k}y^{\alpha_{k}}}dy.$$

To complete, all that remains is to determine the vector $\lambda^{*}.$ For that, one must minimize the dual entropy:

(A5) $$\Sigma \left(\lambda ,\mu \right)=\ln Z\left(\lambda \right)+\sum_{k=1}^{K}\ln\left(e^{d\lambda_{k}}+e^{-d\lambda_{k}}\right)+\langle \lambda ,\mu_{Y}\rangle$$

with respect to λ for each fixed μ. There 〈a,b〉 denotes the standard Euclidean scalar product and μ is the *K*–vector with components $\mu_{k},$ and obviously, the dependence on α is through $\mu_{Y}.$

We mention that when the solution of this dual problem exists, then we have

(A6) $$H\left(g^{*}\right):=-\int_{0}^{1}g^{*}\left(y\right)\ln g^{*}\left(y\right)dy=\Sigma \left(\lambda^{*},\mu \right)=\ln Z\left(\lambda^{*}\right)+\sum_{k=1}^{K}\ln\left(e^{d\lambda_{k}^{*}}+e^{-d\lambda_{k}^{*}}\right)+\langle \lambda^{*},\mu_{Y}\rangle$$

Please note that when d=0, that is, when we suppose that there are no measurement errors, then $\epsilon_{k}=0$ and at $\lambda^{*}$ we have

$$H\left(f^{*}\right):=-\int_{0}^{1}g^{*}\left(y\right)\ln g^{*}\left(y\right)dy=\Sigma \left(\lambda^{*},\mu \right)=\ln Z\left(\lambda^{*}\right)+\langle \lambda^{*},\mu_{Y}\rangle .$$

This can be taken to be the starting point to study the dependence of $g^{*}$ on the sample.

### Appendix A.2. Remarks on the Minimization Process

Let us examine some properties of Σ(λ,μ) related to the minimization process. First, observe that in our set-up, it is a strictly convex function and twice continuously differentiable function, defined on all $R^{K}.$ If we denote by

(A7) $$\phi \left(\lambda \right)=-\nabla_{\lambda }\ln Z\left(\lambda \right)$$

then, when Σ(λ,μ) is bounded below, the first-order condition for $\lambda^{*}$ to be a minimizer is that $\phi (\lambda^{*})=\mu .$ In practice this is not of much help and the minimization of (A5) has to be carried out numerically.

Notice that ϕ(λ) is differentiable and its Jacobian (the negative of the Hessian matrix of ln(Z(λ)) is the negative of the covariance matrix of the fractional powers $y^{\alpha_{i}},$ that is of the positive definite matrix with components $\mu \left(\alpha_{i}+\alpha_{j}\right)-\mu \left(\alpha_{i}\right)\mu \left(\alpha_{j}\right)$ with respect to the maximum entropy density.

Observe as well that (μ) has all its components bounded by 1, thus the image of [0,1] by $\phi^{-1},$ which is continuous due to finiteness of the Hessian matrix, is compact.

To finish, let us note that the quality of the reconstruction is measured by how well the maxentropic density satisfies (9). This follows from the fact that norm of the gradient of (A5) at $\lambda^{*}$ is 0. The explicit computation of (A7) is enough to verify the claim. In the numerical examples described above, the observed value of the norm of (A7) was less than $10^{-5}.$ This was true in almost all the cases. The few exceptions appear as outliers in the computation of the VaR and TVaR described in Section 8.6. This happens mainly when the samples are small and the maxentropic procedure tries to fit a poor estimate of the Laplace transform.

### Appendix A.3. The MEM Method

As the method of MEM is a much less well-known procedure, let us describe the intuition behind it. For further detail, consider [19]. Again, the problem is to solve numerically the integral equation

$$\int_{0}^{1}y^{\alpha_{i}}g\left(y\right)dy=\mu \left(\alpha_{i}\right)$$

with discrete data. As usual, the first step consists of transforming the problem into a system of equation, which turns out to be an ill-posed linear problem, with convex constraints upon the solution. By partitioning [0,1] into *N* sub-intervals, and setting $A_{i,j}={\left(j/N\right)}^{\alpha_{i}}/N$ for i=1,…,K and j=1,…,N. Putting $x_{j}=g\left(\left(2j-1\right)/N\right)$ for j=1,…,N, the transformed problem is

Ax=y

where $y_{i}=\mu \left(\alpha_{i}\right),\;i=1.$ If we consider a small partition into *N* sub-intervals, we already have an ill-posed problem, with $x_{j}\geq 0$ as convex constraint. It is to solve this problem in a way that naturally incorporates the constraints that the method of MEM was developed. Let us denote by $\Omega ={\left[0,\infty \right)}^{N}$ the constraint space and by B its Borel sets. Let $X_{j}:\Omega \to \left[0,\infty \right)$ denote the coordinate maps $X_{j}\left(\xi \right)=\xi_{j}.$ The new problem is now restated as:

$$\mathrm{Find}\;a\;\mathrm{probability}\;\;P\;\;\mathrm{on}\;\;\left(\Omega ,B\right)\;\;\mathrm{such}\;\mathrm{that}\;\;AE_{P}\left[X\right]=y.$$

To make the search for such *P* easy, one starts by putting a reference measure *Q* on (Ω,B) such that supp(Q) equals Ω, and then one looks for a strictly positive ρ(ξ) such that dP=ρdQ satisfies the constraints that is such that

$$AE_{P}\left[X\right]=\int_{\Omega }A\xi \rho \left(\xi \right)dQ\left(\xi \right)=y.$$

The fact that supp(Q)=Ω and ρ(ξ)>0 ensure

$$\xi^{*}=\int_{\Omega }\xi \rho \left(\xi \right)dQ\left(\xi \right)\in \int \left(\Omega \right)$$

That is that all the components of the maxentropic solution $x^{*}$ to our problem satisfy the constraint. It is to determine ρ that the maximum entropy is brought in, and the rest should be routine by now. We direct the readers to [20] for details and further references.

### Appendix A.4. Further Details about the Sample Dependence

To estimate the difference between two densities f(x) and g(x) in the $L_{1}$ norm, the following results are useful. Define

(A8) $$K\left(f,g\right)=\int f\left(x\right)\ln\left(\frac{f(x)}{g(x)}\right)dx.$$

Two properties of (A8) which are important for us here are collected under.

**Proposition** **A1.**
*Consider two densities f and g on [0,∞) and let the Kullback divergence among them be defined by (A8). Then*

(a) K(f,g)≥0 and K(f,g)=0⇔f=ga.e
(b) (Kullback’s inequality) $\;\frac{1}{4}{\left({∥f-g∥}_{1}\right)}^{2}\leq K\left(f,g\right).$

Part (a) is an easy consequence of Jensen’s inequality and part (b) is a guided exercise in [7].

Now, with the notations introduced at the beginning of this section, consider to begin with $K(f_{e},f_{M}).$ Using the representation provided in (A3), definition (A8), and (A6), it takes a simple computation to verify that

(A9) $$K\left(f_{e},f_{M}\right)=\ln Z\left(\lambda_{M}^{*}\right)+\langle \lambda_{M}^{*},\mu_{e}\rangle -\ln Z\left(\lambda_{e}^{*}\right)-\langle \lambda_{e}^{*},\mu_{e}\rangle$$

## References

[1] Gomes-Gonçalves E., Gzyl H. (2014). Disentangling Frequency Models. J. Oper. Risk.

[2] Gomes-Gonçalves E., Gzyl H., Mayoral S. (2015). Maxentropic approach to decompound aggregate risk losses. Insurance.

[3] Feller E. (1971). An Introduction to Probability Theory and Its Applications, Vol. 2.

[4] Panjer H. (2006). Operational Risk: Modeling Analytics.

[5] Lin G.D. (1992). Characterizations of distributions via moments. Sankhya Indian J. Stat..

[6] Jaynes E. (1957). Information theory and statistical mechanics. Phys. Rev..

[7] Kullback S. (1955). Information Theory and Statistics.

[8] Borwein J., Lewis A. (2000). Convex Analysis and Nonlinear Optimization.

[9] Gzyl H. (2016). Sample dependence in the maximum entropy solution to the generalized moment problem. J. Prob. Stat..

[10] Gomes-Gonçalves E., Gzyl H., Mayoral S. (2016). Loss data analysis: Analysis of the sample dependence in density reconstruction by maxentropic methods. Insur. Math. Econ..

[11] Laeven R.J.A., Goovaerts M.J., Melnick E.L., Everrit B.S. (2008). Premium calculation and insurance premiums. Encyclopedia of Quantitative Risk Analysis and Assessment.

[12] Young V., Teugels J.L., Sundt B. (2004). Premium principles. Encyclopedia of Actuarial Sciences.

[13] Rockafellar R.T., Uryasev S. (2000). Optimization of conditional value at risk. J. Risk.

[14] Gzyl H. (2017). Superresolution in the maximum entropy approach to invert Laplace transforms. Inverse Probl. Sci. Eng..

[15] Gomes-Gonçalves E. https://github.com/erikapat/A-review-of-maximum-entropy-methods.

[16] Hyndman R.J., Koehler A.B. (2006). Another look at measures of forecast accuracy. Int. J. Forecast..

[17] Gneiting T., Balabdaoui F., Raftery A.E. (2007). Probabilistic forecasts, calibration and sharpness. J. R. Stat. Soc. Ser. B.

[18] Gomes-Gonçalves E., Gzyl H., Mayoral S. (2016). Maximum entropy approach to the loss data aggregation problem. J. Oper. Risk.

[19] Golan A., Gzyl H. (2002). A generalized maxentropic inversion procedure for noisy data. Appl. Math. Comput..

[20] Gomes-Gonçalves E., Gzyl H., Mayoral S. (2015). Two maxentropic approaches to determine the probability density of compound risk losses. Insur. Math. Econ..

